# Supplementary material for: The discovery of novel noncoding RNAs in 50 bacterial genomes
Source: Nucleic Acids Res. 2024 Apr 22;52(9):5152–65. doi: 10.1093/nar/gkae248 (PMC11109978; doi:10.1093/nar/gkae248)
Supplement: gkae248_Supplemental_Files [file gkae248_supplemental_files.zip › Narunsky 50 Genomes RNA Motifs Files.zip!/Publication/61-Geobacter_sp_M21/Figures/LRC-61-18-NC_012918_1_IGR_67_2945436_2945688.sto.pdf]

LRC-61-18-NC_012918_1_IGR_67_2945436_2945688.sto.r2r

5′-ACCC●CYCCCRGAGGRGAGGR●●A●●G○A●●●●GYY●●YCC●●CUCUGRRUUURRCGC●GUGRC●A○R●●●AA●●UCUGUCA●A●CY●CUY●GUCA●●GAY○●YRGRA●○CA●●AR●YCC●GAAUCAGCAAAAGCCUUUGYC●YUG●CGCCGUUUGCGCAUCCAGUGGCACCUUGUAUACUGCUACCAUUCYCGCUUGCUAUGCCUGUAUUYCAGGYGAACUAAGACCAUAAAGGAGACAGAA
